# Parents can reliably and accurately detect trunk asymmetry using an inclinometer smartphone app

**DOI:** 10.1186/s12891-022-05611-3

**Published:** 2022-08-05

**Authors:** Marie Beauséjour, Delphine Aubin, Carole Fortin, Mohamed N’dongo Sangaré, Mathilde Carignan, Marjolaine Roy-Beaudry, Carolina Martinez, Nathalie Bourassa, Nathalie Jourdain, Philippe Labelle, Hubert Labelle

**Affiliations:** 1 Research Center, Sainte-Justine University Hospital Center, Montreal Québec, Canada; 2 Department of Community Health Sciences, Faculty of Medicine and Health Sciences, Université de Sherbrooke, Campus Longueuil, 150 Place Charles-LeMoyne – Bureau 200, Longueuil, Québec J4K 0A8 Canada; 3 Centre de recherche Charles-Le Moyne, Longueuil, Québec Canada; 4 Department of Surgery, Faculty of Medicine, Université de Montréal, Montreal, Québec Canada; 5 School of Rehabilitation, Université de Montréal, Montreal, Québec Canada; 6 Université du Québec en Abitibi-Témiscamingue, Québec, Canada; 7 Polytechnique Montréal, Montreal, Québec Canada; 8 Orthopedic Division, Sainte-Justine University Hospital Center, Montreal Québec, Canada

**Keywords:** Adolescent idiopathic scoliosis (AIS), Trunk surface asymmetry, Reliability, Validity, Smartphone application

## Abstract

**Purpose:**

An inclinometer smartphone application has been developed to enable the measurement of the angle of trunk inclination (ATI) to detect trunk surface asymmetry. The objective was to determine the reliability and validity of the smartphone app in the hands of non-professionals.

**Methods:**

Three non-professional observers and one expert surgeon measured maximum ATI twice in a study involving 69 patients seen in the spine clinics to rule out scoliosis or for regular follow-up (10-18 y.o., Cobb [0°-58°]). Observers were parents not familiar with scoliosis screening nor use of an inclinometer. They received training from a 4-minute video. Intra and inter-observer reliability was determined using the generalizability theory and validity was assessed from intraclass correlation coefficients (ICC), agreement with the expert on ATI measurements using Bland-Altman analysis, and correct identification of the threshold for consultation (set to ≥6° ATI).

**Results:**

Intra-observer and inter-observer reliability coefficients were excellent ϕ = 0.92. The standard error of measurement was 1.5° (intra-observer, 2 measurements) meaning that a parent may detect a change of 4° between examinations 95% of the time. Comparison of measurements between non-professionals and the expert resulted in ICC varying from 0.82 [0.71-0.88] to 0.84 [0.74-0.90] and agreement on the decision to consult occurred in 83 to 90% of cases.

**Conclusion:**

The use of a smartphone app resulted in excellent reliability, sufficiently low standard error of measurement (SEM) and good validity in the hands of non-professionals. The device and the instructional video are adequate means to allow detection and regular examination of trunk asymmetries by non-professionals.

## Introduction

Adolescent idiopathic scoliosis (AIS) is a 3D deformity of the spine that affects 2 to 4% of the pediatric population [1]. The 3D rotational deformity of the trunk creates a visible posterior protuberance of the ribs and/or of the flank. Identifying and measuring this trunk asymmetry represents a potential for early detection of scoliosis in youth. Nevertheless, scoliosis screening, especially systematic school screening of asymptomatic children as a preventive program, has been the subject of discussion.

The trunk asymmetry can become visually apparent while performing the Adams Forward Bending Test (AFBT), where the child is asked to bend forward at 90^o^, with arms hanging down and head relaxed [2]. The AFBT is a simple and non-invasive screening exam that was used in the initial scoliosis screening programs. It was considered to offer a high rate of detection since the examiners were well-trained to identify even mild trunk asymmetry [3]. As a consequence, many children were sent for orthopaedic evaluation, but many (even up to 80%) did not present with a clinically significant curve (Cobb angle < 11°) and/or never needed treatment [4–7]. In addition, even if spinal braces were largely included in orthopaedic practice, and different reports had demonstrated potential curve stabilization, at that time the available evidence of an effective treatment for the cases detected was considered insufficient [8].

For these reasons, school screening programs using the AFBT as a detection method were not considered to be a cost-effective preventive measure by the Canadian Task Force on the Periodic Health Examination (CTFPHE). This led to a recommendation against scoliosis screening in 1979 [8], and scoliosis screening in schools was officially discontinued in Canada, including in Quebec in the early 1980’s. The same decision was also taken in other countries based on task forces recommendations.

These policy decisions presumably had an impact on the management of patients with progressive scoliosis. Retrospectively studying the referral patterns of suspected cases of AIS in orthopaedic clinics, our team [5, 9, 10] demonstrated that after discontinuation of school screening programs, 20% of patients were referred “late” to a scoliosis clinic to benefit from appropriate and timely conservative management with a spinal brace. Thomas et al. reported that in a US county, the number of referrals to orthopaedic clinics for scoliosis in areas without school screening decreased, as well as the number of spinal brace prescriptions [11].

The Scoliosis Research Society Task Force on Screening (SRS Task Force) conducted a review and re-examined the evidence [12], based on the WHO classical criteria for screening [13]. This report identified the scoliometer (Orthopedic Systems Inc., Hayward, CA) in combination with the AFBT [12, 14] as the reliable and valid tool recommended to measure the external trunk asymmetry. The SRS Task Force also concluded on the effectiveness of the brace treatment, notably from strong evidence provided by a multicenter international trial, the BrAIST study [15]. Similar conclusions regarding use of the scoliometer (adequate evidence that screening tests can accurately detect AIS when used in combination) and the effectiveness of the brace treatment (adequate evidence that bracing may decrease curve progression in adolescents with mild or moderate curve severity) were drawn by the US Preventive Services Task Force in 2018 [16].

The scoliometer is used to quantify the trunk asymmetry, the angle of trunk inclination (ATI), which corresponds to the angle between the horizontal and the plane across the back at the greatest elevation of a rib prominence or lumbar prominence (left and right sides). An ATI between 5 and 7 degrees has been determined as a reference threshold for further medical investigation [12, 17]. Good intra and inter-observer reliability of the scoliometer have been demonstrated in several studies [18–20]. The scoliometer was also shown to improve the specificity of the detection method in comparison to the AFBT alone (for example, 83% [73%-93%] using a scoliometer, in comparison to 60% [47%-74%] with the AFBT alone, for scoliosis curves that were above 20°) [18]. However, the scoliometer is almost exclusively used in orthopaedic clinics and is not accessible to the general public.

**Fig. 1:**
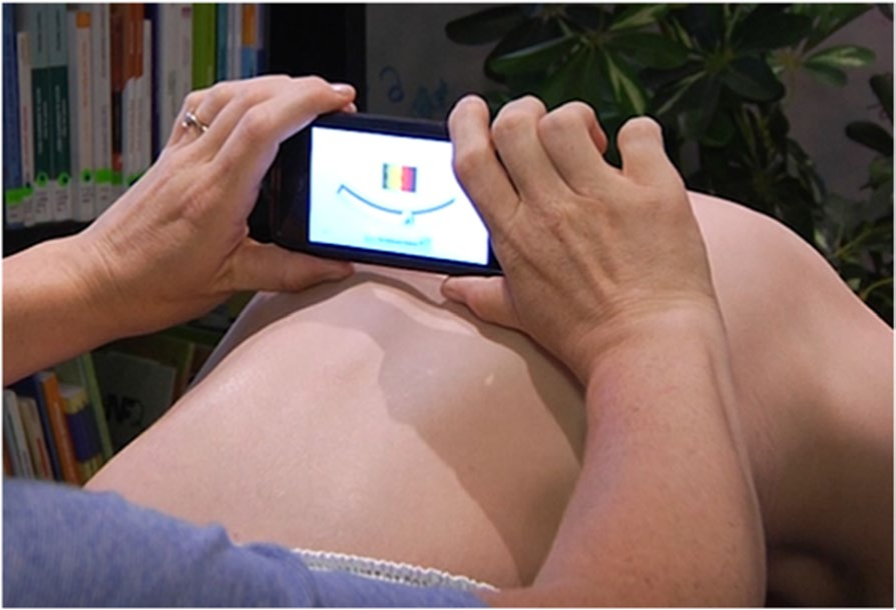
Use of the inclinometer smartphone app as demonstrated in the training video

Thus, alternative solutions are being proposed to facilitate early detection for AIS. The inclinometer applications developed for use on a smartphone appear as a solution to this limited accessibility. Taking advantage of embedded inclinometers enabling angle measurement with a smartphone, these apps reproduce the functions of a traditional scoliometer [2]. They can be used by firmly holding the smartphone between the thumbs and index fingers (Fig. 1), or in combination with a scolioscreen, a device made of medical grade thermoplastic rubber sized to hold a smartphone, and designed to mimic the undersurface of a scoliometer [2].

Previous studies have shown similar reliability and validity between the scoliometer and an inclinometer smartphone app [2, 21–23] but the ATI measurements were mostly taken by health professionals. A systematic review [24] on evaluation methods to screen for back asymmetry supported the reliability of an inclinometer smartphone app in the hands of experimented observers. There is a need to further investigate its value in non-professional users. Given the general accessibility of smartphones, an inclinometer smartphone app becomes an accessible means for primary care providers or non-professionals such as educators and parents to contribute to AIS screening. Such a shared responsibility for scoliosis detection may reduce the number of late referrals to orthopaedic clinics, favor timely initiation of conservative management and, in turn, reduce the risk of surgery [5]. This could also reduce the number of unnecessary referrals to orthopaedic clinics by improving the specificity of the AFBT with an objective measure. Our hypothesis is that non-professionals may reliably and validly measure the trunk asymmetry using an inclinometer smartphone app. Thus, the purpose of this study is to evaluate the intra- and inter-observer reliability and validity of the inclinometer smartphone app in the hands of non-professionals.

## Methods

A sample of 69 young volunteer participants, aged between 10 and 18 y.o., were recruited at the CHU Sainte-Justine orthopaedic clinic between May 2017 and August 2018. These patients were either referred for suspected AIS or followed at the clinic for a confirmed AIS diagnosis.

Three non-professional (but non-familial) observers (adult parents, employees from the CHU Sainte-Justine Research Center without clinical training or education) and one expert orthopaedic surgeon (35 years of experience) were mandated to take ATI measurements from all the participating patients using the inclinometer smartphone app. The three observers were shown a 4-minute training video on the use of the inclinometer smartphone app on the first day of data collection (Fig. 1).

This video created by our research team in collaboration with the educational services of CHU Sainte-Justine describes and demonstrates the procedures to use the inclinometer smartphone app: standard instructions to be delivered to guide patient’s execution of the forward bending test, position of the observer, demonstration on how to slide the smartphone on the child’s back with both thumbs underneath, how to look for the maximum ATI value along the back. The three non-professional observers and the expert measured the ATI from all patients at two occasions. A delay of 15 to 45 minutes separated the two measurement sessions. Patients were encouraged to move and take a few steps between each measurement, as each observer was, in turn, entering the examination room. Assessments were blinded to other observers and the order of measurement was randomly assigned for each patient. The observers and the expert were instructed to record the maximum ATI value measured along the back at each trial. In addition to performing the series of measurements by holding the smartphone between the thumbs and index fingers, one observer and the expert also tested the use of the scolioscreen. In total, for each patient in the sample, twelve measurements of the maximum ATI were taken.

Statistical analyses were performed using the theory of generalizability (G theory) [25, 26] and the Bland-Altman method [27, 28] to assess intra- and inter-observer reliability, as well as the validity of the smartphone app in the hands of non-professionals. The G theory was used to identify sources of variance in the data, and for estimating the proportion of variance explained by the patients (P), the observers (O) and the measurement sessions (S) facets as well as interactions between these facets (PO, PS, OS) and the residual error (POS,e) [26]. We also studied the optimization of the measurement modalities by conducting a “D-study” with either keeping the observer facet fixed (intra-observer) or the session facet fixed (inter-observer). The dependability coefficient (φ) was calculated in these two contexts along with the standard error of measurement (SEM) and the minimally detectable change (MDC= $\sqrt{2}\ast SEM\ast 1.96$, for a 95% confidence interval (CI)) [25].

For intra-observer reliability, we also plotted the differences between the values of the two measurements as a function of the means, for each observer. We estimated the systematic bias (which is the average of the differences among patient’s data) and the proportional bias (which is the slope of the regression line of a Bland-Altman plot) [29].

For inter-observer reliability, we calculated the intraclass correlation coefficient (ICC), with 95%CI, of the form “two-way random effects, single rater, absolute agreement” across the 3 non-professional observers. Finally, the validity assessment relied on the Bland-Altman method comparing the measurements for the first observation of the expert with those of each of the non-professional observers, as well as agreement in the correct identification of patients with ATI ≥6^o^.

All participants and/or their parents as well as non-professionals signed informed consent/assent forms, and the project was approved by the ethics committee of CHU Sainte-Justine. Statistical analyzes were carried out with Genova software [30] and IBM SPSS Statistics for Windows (Version 25.0. Armonk, NY: IBM Corp.).

## Results

The study sample was composed of 17 boys and 52 girls. The mean age was 14.2 years old (standard deviation (SD)=1.6). The Cobb angle of the main scoliosis curve varied between 0^o^ and 58^o^ in the patients. For 39 patients, the Cobb angle was less than 20^o^, and for 30 patients, it was equal or greater than 20^o^. The mean ATI (as measured by the expert) was 6^o^ (SD=4^o^), and 28 patients had an ATI equal to or greater than 6^o^.

The study of the components of variance from the G theory [26] identified the inter-patient variance as the main source of variance (82% of total variance). The variance component associated with the observers was low, 1%, while 3% of the variance came from the interaction between the patients and the observers (PO). The variance attributed to the sessions was 0%, as well as the interaction between the observers and the sessions (OS). The variance associated with the interaction between the patients and the sessions (PS) was also low at 2%. However, the interaction between the patients, the observers and the measurement sessions, explained 11% of the variance.

### Intra-observer reliability

In the D-study (observer-fixed design), the intra-observer reliability was excellent (ϕ = 0.92) and the SEM was 2.1° for one ATI measurement taken, but decreased to 1.5° if two measurements were taken by the same observer. Thus, when taking two measurements of the ATI, a non-professional may detect a change of 4° between examinations 95% of the time.

**Fig. 2:**
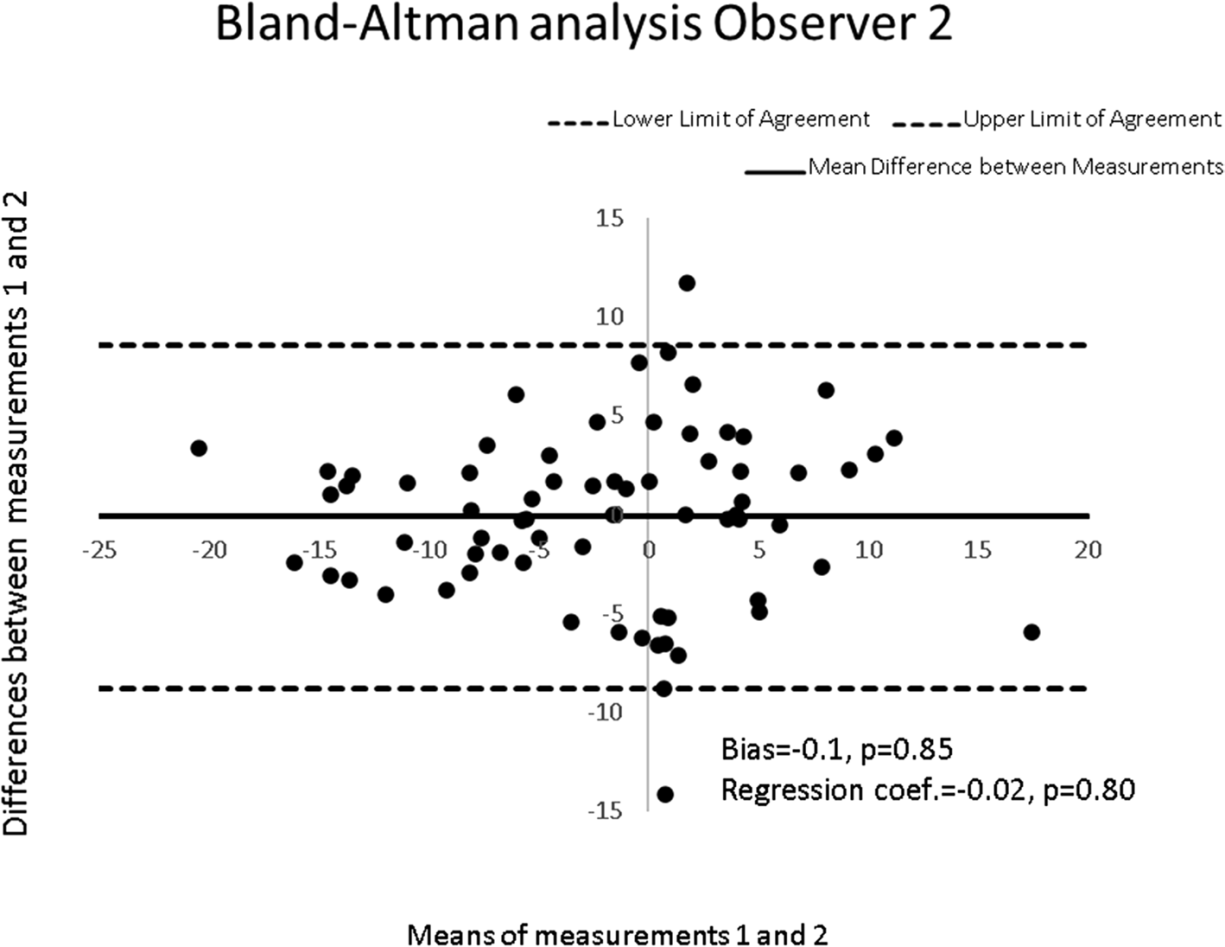
Bland-Altman analysis for observer 2

The Bland-Altman method revealed no statistically significant bias for intra-observer measurements when the smartphone is firmly held between the thumbs and index fingers or used in combination with the scolioscreen, both for the non-professional observers and the expert, with biases between 0.1° and 0.4°. The smallest bias (0.1°) was obtained by the expert using the thumbs and index fingers. There was also no proportional bias for all observers and for the expert in the two measurement conditions, with regression coefficients between 0.02 and 0.07. A typical plot is presented in Fig. 2.

### Inter-observer reliability

In the D-study (session-fixed design) the inter-observer reliability was also excellent (ϕ = 0.92) with a SEM of 2.1° for one measurement. For two measurements taken by, for example, the two parents of a child, the SEM would reduce to 1.5°. They would be able to detect a 4° difference in measurements 95% of the time. The overall intraclass correlation coefficient for the 3 observers was: 0.88; 95%CI [0.82-0.92].

### Validity

A statistically significant systematic bias (slight overestimation of 0.8° and 1.1°) was identified for 2 of the 3 observers when compared to the expert while the smartphone is used firmly held between the thumbs and index fingers. A proportional bias was also identified when the first observer is compared to the expert while the smartphone is used in combination with the scolioscreen.

**Fig. 3:**
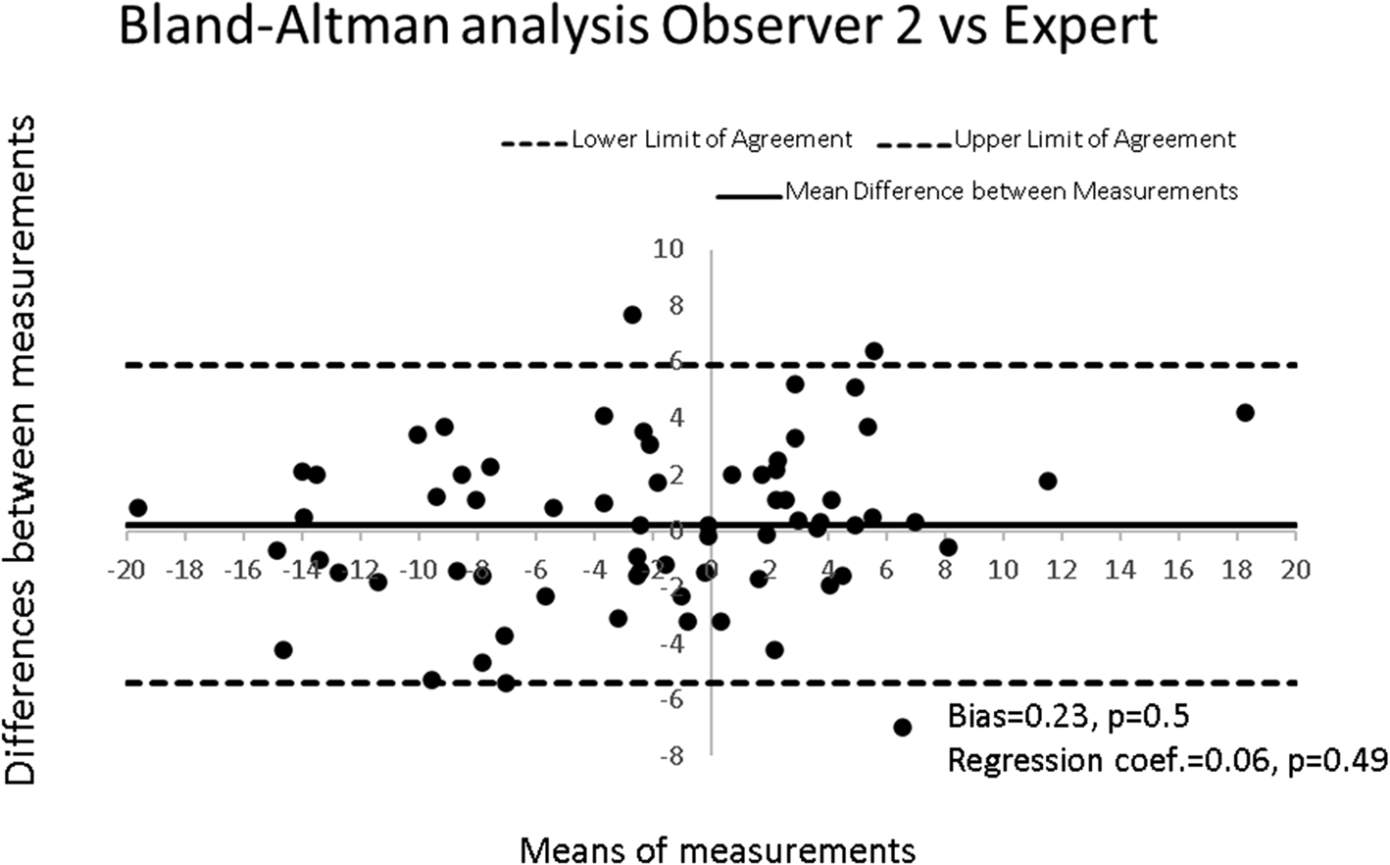
The Bland-Altman analysis for observer 2 in comparison to the expert

Comparison of the measurements taken by the non-professionals and the expert led to ICC ranging from 0.82 [0.71-0.88] for observer 1, 0.84 [0.74-0.90] for observer 2, to 0.84[0.73-0.89] for observer 3. Thus, an agreement between non-professionals and the expert on the identification of the threshold to seek medical advice (ATI ≥ 6 °) was reached in 83% to 90% of cases (Fig. 3).

## Discussion

In the literature, the reliability and validity of an inclinometer smartphone app used among non-professionals remained an unanswered question [24]. In this study, we investigated the intra and inter observer reliability by two methods: the G theory and the Bland-Altman analysis.

The results obtained in this study demonstrated excellent intra-observer reliability. Systematic biases between two measurements taken by the same observer were not statistically significant in all cases, and these mean differences were all below the clinically acceptable threshold of 0.5° average difference for ATI that was consensually a priori established by our team [2]. The results were even slightly improved when the first observer used the smartphone in combination with the scolioscreen. In this study, no difference was observed in the expert reliability assessments with or without the scolioscreen. There was also no proportional bias, meaning that the error was very stable between measurement sessions, regardless of the magnitude of the measured ATI, and especially with the use of the scolioscreen.

The results from the G theory also confirm that the intra-observer error is low, with variance O=1% and variance S=0%. Plausible explanations for the PO variance of 3% may come from some variability in the instructions for AFBT that were given to the patient, as well as from the relative height and shape of the patients and observers. The variance component related to the interaction POS at 11% may come from the non-standardized elements of the protocol, such as patient movement or fatigue, and distraction.

Inter-observer reliability was excellent from the results of the D-study. Mean differences between observer measurements were also lower than the 2° average difference for ATI that was consensually a priori established by our team as suitable for detection in primary care settings or family use.

The validity was satisfactory to recommend the use of the inclinometer app as a detection tool in non-professionals. Systematic biases were below the clinically acceptable threshold of 2° average difference between measurements taken by a non-professional observer and those taken by an expert surgeon, for both measuring conditions (with and without the scolioscreen). There was also a significant proportional bias for one of the comparisons (observer 1 vs expert when using the scolioscreen). However, the regression coefficient was very small (0.15), indicating that the proportional bias for this comparison did not have a serious impact on the results. Agreement between non-professional observers and the expert on the identification of an ATI ≥ 6° is satisfactory. Our study results indicate that non-professionals, such as parents, may use the inclinometer smartphone app to reliably detect a significant rib/flank hump and seek medical opinion and clinical assessment for appropriate use of medical resources.

Previous studies of reliability and validity comparing the scoliometer and a smartphone app have been carried out in experienced observers [21–23]. For example, the study by Qiao et al. with 64 patients compared the scoliogauge smartphone application and the scoliometer. All measurements were performed by surgeons. The study found an overall intra-observer ICC of 0.954 for the scoliometer and 0.965 with the app. The overall interobserver ICC was 0.943 for the scoliometer and of 0.964 with the app [21]. It is interesting to note that Qioa et al. found a lower ICC = 0.819 for small curves; in our study, the variability was stable among ATI values. In another study from Québec, Canada, Balg et al. showed excellent results with a smartphone application in comparison to the scoliometer: no systematic bias and 95%CI of ±4.4°. In their study, carried out with 34 patients with AIS and whose measurements were taken by healthcare professionals (without an adaptor such as the scolioscreen), the intra and inter observer ICC were respectively 0.961 and 0.901 [31]. The study by Driscoll et al., a rare study involving a non-professional observer, showed similar results. In fact, in 39 patients with AIS, the authors showed good intra observer reliability ICC=0.89 and satisfactory inter-observer reliability (lower than in the current study) with ICC=0.75 using the smartphone alone and 0.89 using the scolioscreen [2].

A plausible hypothesis to explain better performance of the non-professionals in the current study than what was expected from a previous study [2] may reside in the use of a standardized training in the form of an educational video as corroborated by the low variance associated with observers (1%). This study also differs from the study of Driscoll et al. in its results demonstrating that reliable and satisfactorily valid measurements can be obtained even without the use of the scolioscreen device (although the results are improved by the use of the scolioscreen). This makes it even easier and more convenient for non-professionals to start using the smartphone app.

This study demonstrated that non-professionals would be able to learn how to manipulate an inclinometer smartphone app to properly follow-up on a child’s trunk asymmetry. This is different from several previous studies where results were available for trained experts only [21, 23, 31]. In addition, non-professional observers in the current study measured all participating patients (n=69) as opposed to non-professional observers in the study from Driscoll et al. [2] who have only measured their child. One may hypothesize that the observers in this study have improved their performance over time. It is important to consider however that the three non-professional observers did not receive any feedback on their measurement techniques. They could have changed their methods over time or gained confidence in what they were doing but they were never told if their technique was adequate. We compared the results obtained on the first versus the second half of the data collected and no temporal trends was found in the data.

The use of an educational video has the advantage to provide standardized directions and visual demonstration of the technique. It may be paused or repeated for best comprehension. Interviewed observers confirmed that the self-training was appropriate and considered sufficient to perform the ATI measurement after two views of the video, as corroborated by the low sources of O and S variances and of the interactions PO, PS. This training method facilitates the dissemination and wide use of the tool by primary health care providers, physical educators and parents who would be interested to monitor the trunk asymmetry in a child.

This study has some limitations. One potential bias comes from the experimental set-up where observers were not blind to their own measurements. Even if several minutes separated the two measurements from a given observer with a given patient, and that more than one patient were included in the study from the same half day of clinic, often interleaved, there is a possibility that the observer remembered the first measurements and adjusted his/her observation to match the second measurements. However, the observers were not aware of the objectives and hypotheses of the project. Interviewed observers said that this bias was unlikely since they needed to remain concentrated on the good quality of measurement at each occasion and they were more preoccupied with doing the task properly than trying to “cheat” or to copy their previous result. Our methodological choice to rely on a full design (all patients are measured by all observers) allowed us to generalize our results to the “universe” of similar non-professionals, according to the G theory. As previously mentioned, this may have caused the observers to improve during the study. But the available data do not support a learning curve trend, probably mostly because the observers never received feedback on their execution of the technique, and they were blind to other observers’ (including the expert’s) measurements. Another possible bias in this study concerns the period of patient recruitment which lasted more than a year. However, in this study, the samples from two different time periods in 2017-2018 were compared and no significant differences (data not shown) in the reliability and validity results were identified over the study period. We acknowledge the lack of comparison with the non reference standard, the scoliometer, in this same study. As mentioned, this comparison was shown to be appropriate in several previous studies. Our research question was not to duplicate this comparison but to investigate the reliability and validity of the tools in the hands of non-professionals. Doing all these measurements and comparisons in the same study would have inappropriately increased the burden for the participants, who already had to execute the AFBT twelve times.

The personal skills of the observers may also have influenced the results, as some people are naturally more skilled in handling an electronic device. However, our results indicated that these differences appear negligible and may not have an impact on the use of the smartphone app. Finally, since the patients in the sample in this study were not the children of the observers, the observers may have felt less comfortable taking measurements of the ATI than would be the case for their parents. There is an added value of confirming the measurements with a second observer (for example, the two parents) and/or encouraging the observer to repeat the measurements twice. According to the D-study, the recommendation is to take the average of two measurements recorded by the observers at two sessions separated by a 15 minute pause to get the best evaluation, and to set a 6-degree ATI threshold. Our study shows that when taking 2 measurements of the ATI, a non-professional may detect a change of 4° between examinations 95% of the time. This means that an observed variation between two measurements could be associated with a certain progression of the curve. It could be used at home to follow-up on potential changes in the trunk asymmetry between scoliosis management visits, or for periodic evaluation of mild/non clinically significant curves that were discharged from clinic.

Finally, the question about the value of school scoliosis screening program is beyond the scope of this study. As described elsewhere [12, 32, 33], classical criteria were established to assess the effectiveness of a screening program. They have recently been reviewed and discussed in light of public health concerns such as coordination of the program components, impacts on the healthcare system, as well as societal acceptability. In particular, in a recent systematic review followed by a Delphi expert consensus protocol [34], twelve consolidated principles for screening were elaborated. The present study contributes to *principle* #4 *Screening test performance characteristics,* looking at the key components specific to the test: accuracy and reliability. A limitation of the current study is that it does not contribute much to the evidence about the benefits to harms ratio of screening for trunk asymmetry, notably in terms of health services overuse. Research evidence on this aspect was considered insufficient in the most recent report from the US Preventive Services Task Force [16]. In addition, this observation suggests, as per *principle #6 post screening test options* [34], that health care pathways for children with positive screening tests should be carefully examined and properly evaluated before elaborating recommendations for test implementation.

## Conclusion

This study demonstrated the intra- and inter-observer reliability and the validity of an inclinometer smartphone app when used by non-professionals. Non-professionals could learn to use the inclinometer smartphone app with a training video in order to take reliable and valid measurements of a child's trunk asymmetry. Thus, with a reference threshold of 6°, a non-professional would be able to make an early detection and favor timely and appropriate medical assessment of back deformities.

## Data Availability

The datasets generated, used and analysed during the current study are available from the corresponding author on reasonable request.
